# Measles vaccine coverage among children born to Somali immigrants in Norway

**DOI:** 10.1186/s12889-021-10694-z

**Published:** 2021-04-07

**Authors:** Sherin Marie Jenness, Preben Aavitsland, Richard Aubrey White, Brita Askeland Winje

**Affiliations:** 1 Institute of Health and Medicine, University of Oslo, Oslo, Norway; 2 Division of Infection Control and Environmental Health, Norwegian Institute of Public Health, Oslo, Norway

**Keywords:** Measles, MMR vaccination, Vaccine coverage, Somalia, Immigrants, Vaccine hesitancy

## Abstract

**Background:**

Despite overall good vaccination coverage in many countries, vaccine hesitancy has hindered full coverage and exposed groups to the risk of outbreaks. Somali immigrant groups have been known to have low measles vaccination coverage, leading to outbreaks in their communities. Current research indicates a general lack of trust in the healthcare system, the use of alternative information sources and inadequate health literacy can be contributing factors. We explore measles vaccine coverage in children born to Somali parents in Norway, whether it has changed over time and factors that may influence coverage.

**Methods:**

Data was extracted from the National Population Register on all children born in Norway from 2000 to 2016, where both parents originated from Somalia. Date of birth, gender, residential area at birth and date of immigration and emigration for both parents was linked to information on measles vaccination from the National Immunisation Register.

**Results:**

We found that children born to Somali immigrants in Norway had suboptimal measles vaccine coverage at 2 years; for children born in 2016 the coverage was 85%. Coverage declined between 2000 and 2016, and at a greater rate for boys than girls. Children born to mothers residing in Norway for 6 years or more had lower coverage compared to those with mothers residing less than 2 years prior to their birth. Children born in the capital and surrounding county had significantly lower coverage than children born elsewhere in Norway.

**Discussion:**

New targeted interventions are needed to improve measles vaccine coverage among Somali immigrants in Norway. Some possible strategies include using Somali social media platforms, improving communication with Somali parents and tighter cooperation between various countries’ vaccination programmes.

## Background

An increase in measles outbreaks in Europe in 2016–2019 has been attributed to a decline in vaccination coverage, partly due to vaccine hesitancy. While in 2007 fourteen countries in Europe reached WHO’s stated goal of a 95% measles vaccination coverage for two doses, only four countries did so in 2017. If suboptimal vaccination coverage persists, there will continue to be a high risk of widespread measles circulation in Europe [1].

The situation in Europe appears to be a part of a global trend. In 2019, the world experienced the highest number of measles cases since 2006. Although the largest outbreaks occurred in countries with low measles vaccination coverage, outbreaks also occurred in countries with high national coverage. This can be explained by gaps in the vaccination coverage in subgroups of the population sharing a common community, age, or religious affiliation [2].

We are aware of three measles outbreaks affecting the Somali diaspora in Western countries in the last decade. The largest outbreak in Norway since 1997 was centred in an under-vaccinated Somali community in Oslo. It started in January 2011 with an importation case from Ethiopia. The virus then spread to 18 cases mainly through unvaccinated Somali children and later via emergency clinics to unvaccinated Norwegian children, five of whom were under the recommended age for the first dose of MMR vaccine [3]. Subsequent discussions with the Somali community revealed that under-vaccination was associated with the belief that MMR vaccine causes autism [4].

The other two outbreaks were located in Minnesota. In 2011, an unvaccinated US-born Somali child was infected while visiting Kenya, leading to an outbreak with 21 cases [5]. In 2017 there was a second outbreak, eventually becoming Minnesota’s largest since 1990. Of the 75 reported cases in this outbreak, 91% were unvaccinated and 81% were of Somali decent. At the time of the outbreak, the measles vaccination coverage amongst 2-year-old Somali children born in Minnesota was as low as 42% [6]. As in the Norwegian case, parents in the Minnesotan Somali community had also voiced concerns about MMR causing autism [6, 7].

Since 1983, the MMR vaccine has been offered free to all children living in Norway at age 15 months and 13 years through municipal health clinics and schools [8]. In 2018, Norway had an overall coverage of 96% for the first dose and 93% for the second dose [9, 10]. Although Norway is considered to have eliminated measles [1], imported cases can still lead to outbreaks if clusters of unvaccinated individuals exist, as the above outbreaks demonstrate.

Somalis are the largest non-western immigrant population in Norway and the 5th largest immigrant population overall. They number almost 29,000 individuals which is approximately 0.5% of the total population, not including their Norwegian-born offspring, [11]. Inadequate health literacy and underutilization of preventive services among Somali women in Norway has been reported [12, 13]. Based on these factors and previous measles outbreaks, we investigated measles vaccine coverage in children born in Norway to Somali parents and examined factors that are associated with non-vaccination in order to guide future studies and targeted measures to improve coverage in this group.

## Methods

### Data and linkages

We obtained data from the National Population Register on children born in Norway from 2000 to 2016 for whom both parents originated from Somalia (*n* = 11,600). This data included date of birth, gender, residential area at birth and the last registered dates of immigration (and any later emigration) for both parents. We obtained information on measles vaccination (date of vaccination and vaccine-type) from the Norwegian Immunisation Register (SYSVAK [14]) for these children at 2 years of age, i.e. from 2002 to 2018. The data sets were linked using the 11-digit personal identifier provided to all Norwegian residents, and de-identified by an external partner before the researchers were given access.

Eligible children were those born in Norway from 2000 to 2016 and, who remained resident in Norway until the end of the calendar year in which they turned two. We excluded from the study 266 children who emigrated before the end of this observation period, leaving 11,334 in the analysis.

We calculated age at vaccination as the difference between date of vaccination and date of birth. We calculated mother’s length of residency in Norway prior to the child’s birth as the number of days between the mother’s registered date of immigration and her child’s date of birth. The immigration date was missing for 13 mothers. For 1283 (11%) mothers, the immigration date was later than the child’s date of birth in Norway. Possible explanations are: (i) that the mothers had emigrated and then immigrated again later (as we had the last date of registration), (ii) that they arrived in Norway as asylum seekers and resided in Norway a period before they were granted residence and registered in the National Population Register, or (iii) an error in registration. These dates were recoded to missing.

To calculate length of residency in Norway we used information from the mothers whenever this was available. If the mother’s date of immigration was missing, we used the father’s date of immigration. In 411 children (3.5%) information from both parents was missing. We categorized length of residency in Norway as: < 2 years (< 730 days), 3 to 5 years (≥730 days to < 1825 days) or ≥ 6 years (≥1825 days).

We defined residence as the county in which the children were born, and divided this into three categories: Oslo (the capital), Akershus (the suburban county surrounding Oslo), and Norway other (elsewhere in Norway).

### Vaccination status

We defined measles vaccine coverage consistent with published national coverage estimates in Norway, i.e. the proportion (with 95% confidence intervals) of children who had received at least one dose of measles vaccine during the observation period, i.e. between 12 months of age and the end of the year in which they turned two.

Children classified as unvaccinated were: children without measles vaccines registered in SYSVAK (*n* = 1437) by the end of the calendar year in which they turned two, or children who had received one dose of measles vaccine before 12 months of age, but no additional dose before the age of two (*n* = 42).

### Regression analysis and effect modification

We ran a multivariable linear regression [15] to estimate the associations (as percentage point differences) of the outcome (vaccinated versus non-vaccinated) with the following covariates: year of birth (2000 to 2016), gender (male/female), mother’s length of residency in Norway (< 2 years, 3 to 5 years, and > 6 years), and residential area (Oslo, Akershus, and Norway other).

We investigated whether there was a significant interaction between the yearly trend and (i) gender, (ii) mother’s length of residency in Norway, (iii) residential area, (iv) gender and mother’s length of residency or (v) gender and residential area, by adding interaction terms between yearly trend and the aforementioned variables. Our final model included an interaction term between the yearly trend and gender.

## Results

In total 11,334 children born 2000–2016 were eligible for the study, 9855 (87%) were vaccinated and 1479 (13%) were unvaccinated.

**Fig. 1:**
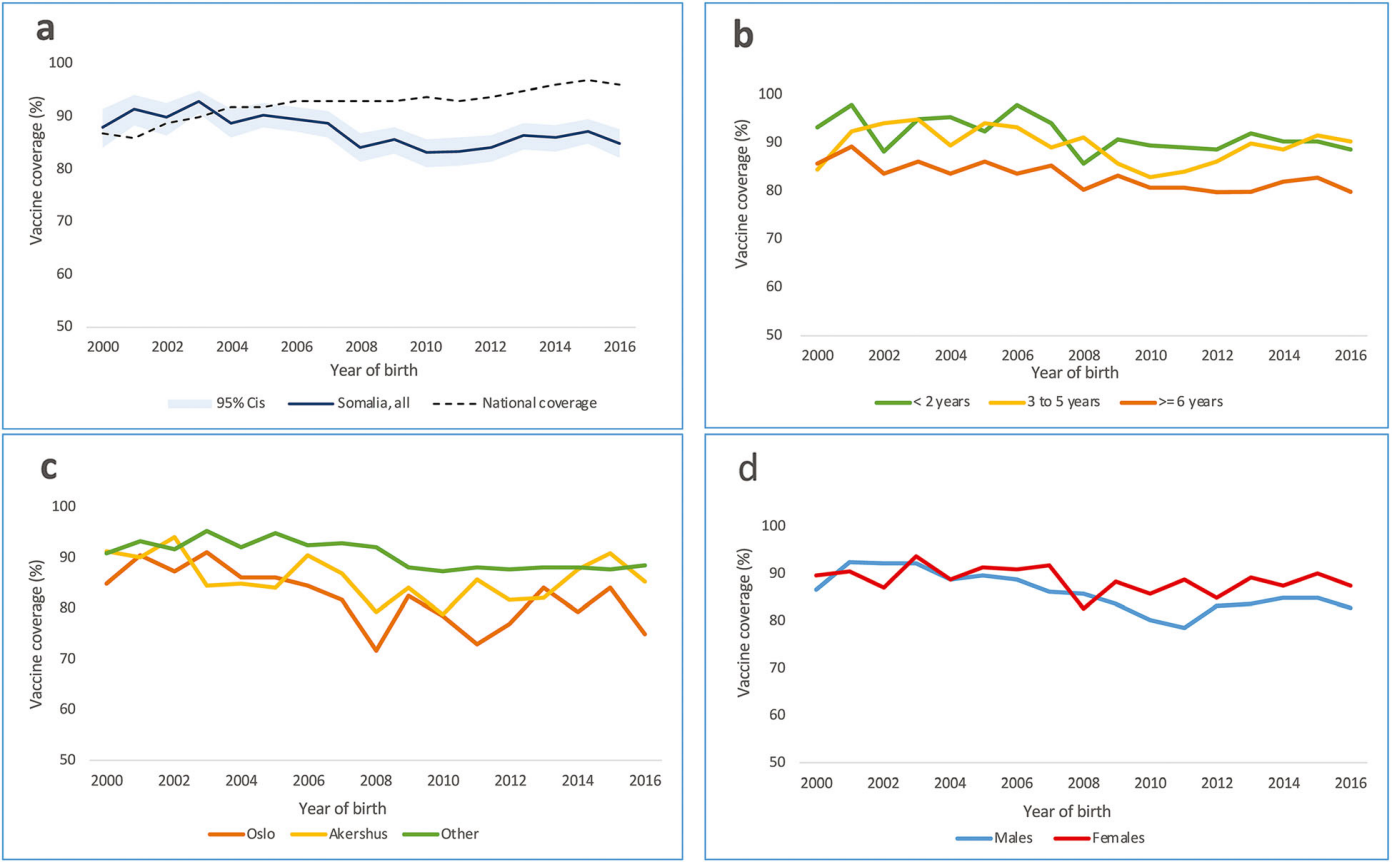
Measles vaccine-coverage at 2 years for children born in Norway from Somali immigrants, years of birth 2000–2016 (*n* = 11,334), (**a**): overall, (**b**): by mothers time in Norway prior to the child’s birth, (**c**): by residential area at birth, and (**d**): by gender

Children born in Norway to Somali immigrants had lower measles vaccine coverage than the national average and this discrepancy increased over time. Whereas the national average slowly increased, the coverage in the Somali group showed an initial increase, followed by a decrease and then an 8-year plateau (Fig. 1a). For children born in 2016, coverage was 85%. The national average was 96%.

After excluding 411 children due to missing information on mother’s length of residency in Norway, our regression model (Table 1), showed that mother’s length of residency was negatively associated with vaccine coverage (*p* < 0.001). Children, whose mothers had resided in Norway for 6 years or more had significantly lower coverage compared to those whose mothers had arrived within the last 2 years prior to delivery. We found no significant difference in coverage between children of mothers with less than 2 years residency compared to 3 to 5 years residency (Table 1, Fig. 1b).

**Table 1:** Difference in measles vaccination coverage among children born in Norway to Somali immigrants, 2000-2016

Covariates	Group size n (%)	Percent point difference in coverage	95% confidence intervals	***p***-value
Mother’s time in Norway				
< 2 years	2534 (23)	Reference		
3 to 5 years	3463 (32)	−1.0	− 2.7 to 0.7	0.252
≥ 6 years	4926 (45)	− 6.7	−8.4 to − 5.0	< 0.001
Residential area				
Norway, other	6015 (55)	Reference		
Oslo county	3230 (30)	−6.5	−8.0 to −4.9	< 0.001
Akershus county	1678 (15)	−3.4	−5.2 to −1.6	< 0.001
Gender - birth year interaction				
Girls^a^		Reference		
Boys born in 2000	160 (54)	0.8	−2.0 to 3.6	0.571
Boys born in 2004	248 (47)	−0.9	−2.8 to 1.0	0.373
Boys born in 2008	370 (50)	−2.5	−3.8 to −1.2	< 0.001
Boys born in 2012	396 (49)	−4.2	−5.6 to −2.7	< 0.001
Boys born in 2016	398 (51)	−5.9	−8.1 to −3.6	< 0.001
Birth year (trend, showing difference per year)				
Girls	5282 (48)	−0.2	−0.4 to − 0.0	0.022
Boys	5641 (52)	−0.7	−0.8 to − 0.5	< 0.001

^a^*Girls born in the corresponding year*

Multivariable linear regression to calculate the difference in measles vaccination coverage among children born in Norway to Somali immigrants, 2000-2016, by year of birth, gender, mothers’ time in Norway and residential area. Mothers’ time in Norway was missing for 411 children. These were excluded from the regression analysis, n=10923

Measles vaccine coverage was significantly associated with the area in which children were born (Oslo, Akershus, or elsewhere in Norway) (*p* < 0.001). Children born in Oslo and Akershus counties had significantly lower coverage than those born elsewhere in Norway (Table 1, Fig. 1c).

The trend in yearly vaccine coverage was significantly modified by gender. We found a significant annual percentage points reduction in vaccine coverage for boys (− 0.7, [95%CI − 0.8 to − 0.5]) over time, i.e. children born in more recent years had lower coverage. This reduction was less severe for girls (− 0.2 [95% CI − 0.4 to − 0.0]) (Table 1, Fig. 1d). We also expressed this time-gender interaction as the difference in coverage between boys compared to girls at different years of birth (Table 1). We did not find any other significant interactions.

## Discussion

We found that children born to Somali immigrants in Norway have suboptimal measles vaccine coverage. For children born in 2016, coverage was 85% which is lower than the 96% national average, and more importantly it is below the 95% threshold recommended by WHO. Coverage declined between 2000 and 2016, and at a greater rate for boys than girls. Children born to mothers residing in Norway for 6 years or more had lower coverage compared to those with mothers residing less than 2 years prior to their birth. Children born in the capital and surrounding county had significantly lower coverage than children born elsewhere in Norway.

### Findings in context

The Somali immigrant community has a significantly lower than average measles vaccine coverage, and statistics collected in association with this study found this community to be unique in this respect. Although some other immigrant populations have lower measles vaccine coverage than the national average, none showed persistent lower coverage over time. Most groups had coverage at the level or above the national coverage and no other population showed significant gender differences (data not shown). Somali immigrants are therefore a group in need of extra consideration when planning future immunisation campaigns, if Norway is to maintain its elimination of measles.

The annual decline in vaccine coverage was more severe for boys than girls. This gender difference implies omitting MMR vaccination involves some level of intentionality. That is, general factors such as availability, religious motives, or attribution to “poor integration” should affect both genders equally. This statistic, coupled with the fact that autism is more prevalent in boys [16], supports qualitative research linking fear of autism to MMR refusal. As one Somali mother in the UK explained, “Just my daughter she’s used the MMR … I see that many families, Somali family, I see that the MMR the problem of the boys, not the girls...” [17].

Longer residency time does not seem to improve vaccine uptake among Somalis in Norway. This was an unexpected finding since one might assume immigrants would increasingly adopt the health-seeking behaviour and values of the society around them. Since current national strategies do not seem to be entirely effective in this community, more tailored approaches may be needed.

In the capital, Oslo, and its surrounding suburbs, measles vaccination coverage is lower compared to elsewhere in Norway. Since qualitative studies have found that mothers face peer pressure when considering vaccination [18], it is possible that this pressure is felt more strongly in a larger Somali community such as in Oslo. In addition, a larger local Somali network may be more self-reliant and therefore enable a greater isolation from the rest of society. This may hinder trust and information exchange with healthcare providers [18]. A qualitative study of influence and information flows within Somali social networks would be helpful in clarifying the reasons behind this finding.

### Comparison to other studies

Although the discrepancies we found are smaller, our results support other studies in western countries that found low measles vaccination coverage among Somali immigrants. In Minnesota coverage varied according to the mother’s country of origin, with children born to Somali mothers having the lowest—only 44.2% [19]. In Washington state, children born to Somali parents were less likely to be immunized against measles, while there was no difference between them and children of US-born parents when comparing other vaccines [20]. In Birmingham, UK the Somali community represents a growing population with some of the lowest levels of preschool immunisation [17]. Districts in Stockholm with high percentages of Somali immigrants also reported MMR coverage at around 70–72% compared to a national average of 97% [18].

Several studies have explored reasons given by Somali mothers for not vaccinating. These include issues involving vaccine safety, vaccine efficiency and experiences with healthcare providers [17, 18, 21]. One study also mentions the porcine gelatine present in most MMR products as a religious barrier to vaccination [17]. However, the most common reason given for MMR vaccine hesitancy is a perceived risk of autism. Somalis are more likely than non-Somalis to believe that autism is caused by vaccines [21]. Although Somali mothers are generally positive towards immunisation, they make a distinction between MMR and other preschool immunisations [17, 18]. In our study-population, the same children had pertussis vaccine coverage comparable to the national average. As mentioned earlier, the apparent intentional MMR avoidance we found in Norway may also be linked to these autism concerns. Further qualitative studies are needed to clarify this issue.

Somalis are a “truly globalized nation”. An estimated 14% of Somalis live outside of Somalia’s national borders as a large and influential transnational diaspora [22]. Although tight-knit Somali communities are scattered around the globe, they maintain relationships with family and friends in both their homeland and other countries. Many have a hope of returning 1 day to Somalia, and therefore watch Somali news or follow diasporic media to stay up to date with the situation back home [23].

These information flows also influence health-seeking behaviour locally. When making the decision to vaccinate, Somali mothers face peer pressure not only from friends and family, but also from those they don’t know personally. Stories circulating in the local community can affect mothers’ confidence in vaccine safety and create a perception that they are putting their child at risk if they choose to vaccinate [18]. In Sweden, some mothers choose to skip information meetings at child healthcare centres and rely on receiving information from mothers that attended rather than getting advice directly from nurses [24]. Information transfers are not just limited to local networks but flow across the transnational Somali diaspora. An example of this is a well-known Somali psychiatrist living in Norway whose role has attained a transnational character. It appears his global influence is only limited by his capacity and time, as he explains:

“People will find out where there is a Somali doctor and seek contact. Since I was one of the first Somali GPs in Norway, many know me, and many Somalis contact me from countries like Denmark, Sweden, and Britain. Everyone who has been in contact with me, convey his or her knowledge of me to others. Also, if someone knows my wife or our children, then they will contact me.” [25]

Our graph showing the decline in vaccine coverage amongst Somalis in Norway appears to correlate over time with similar graphs published from research in Minnesota [26]. If this is the case, it indicates that the decisions of Somali parents locally are in sync with those of the global Somali diaspora.

### Limitations of this study

The calculated mother’s length of residency in Norway may not be entirely accurate, as registration of immigration may not correspond exactly with actual time of arrival in Norway. In some cases, the father’s date of immigration was used.

If children are vaccinated outside Norway and do not report this to their health care providers then SYSVAK will not be updated, and the data will show lower than actual vaccination coverage. Somalis are known to seek medical advice and services when they visit family in the horn of Africa [27]. It is therefore possible that some children get vaccinated during their visit, though it is unlikely that this has impacted our study to a significant degree, specifically since coverage for pertussis vaccination was at the same level as the national average for the same group of children (unpublished data).

Our study includes only second-generation immigrant children born in Norway of two Somali parents. This potentially excludes a group that may be a part of the wider Somali immigrant community but do not meet these criteria. These may include children of mixed heritage with one Somali parent, or third generation children, for example. Several studies focus on newly arrived immigrants. However, we have excluded this group since our method using data from national registries is unable to distinguish between missing registrations (lack of post-registration upon arrival in Norway) and lack of vaccination.

Studies have shown that Somali parents postpone the MMR vaccine until their child starts to speak [17, 18]. We were unable to disentangle delay in vaccination and vaccine refusal. Vaccine coverage may have improved if measured at a higher age.

Our study is based on registry data and is statistical by nature. The questions it raised were explored though reference to other published studies. Future quantitative and qualitative studies are required to fully understand the issues and potential solutions.

### Implications

The low measles vaccination coverage amongst Somali immigrant communities globally has been observed for a few years now. Several countries have implemented targeted vaccination campaigns, but have they achieved their goals? Following the 2011 outbreak in Norway, targeted campaigns managed to vaccinate 25 Norwegian-Somali children [4]. Although attention was brought to this vulnerable group, our results show that coverage did not improve long-term.

Due to globalisation, network-based strategies may prove to be more effective than top-down national strategies [28]. If the “information” about MMR vaccines circulated in Somali transnational networks is persistently negative, local short-term campaigns will have little long-term impact. The global is affecting the local and so local campaigns need to have a global perspective i.e. a “glocal” approach.

Social network analysis may be a helpful tool here, to determine influencers in the Somali community at both a local and global level. Empowering trusted figures in the Somali diaspora can leverage their influence and lower Somali hesitancy globally. For example, someone like the aforementioned Somali psychiatrist could be equipped with help and resources to set up a healthcare website for Somalis. The website could give good advice on vaccination, contraception, mental health and other relevant health issues for Somalis, and would potentially have a global impact. Conversely, empowering a key Somali Youtuber in Minnesota, by showing their videos in waiting rooms, for example, may have a positive impact locally in Norway.

Although multiple studies have failed to find any link between MMR vaccination and autism, a decrease in MMR vaccination has been observed in Somali communities following a study revealing a higher prevalence of autism amongst Somalis [29]. The Somali-Minnesotan community was visited several times by Andrew Wakefield, who was struck off the medical register in the UK in 2010 for deliberately falsifying research to suggest that the MMR vaccine caused autism [30, 31]. Today, it is difficult to talk about the MMR vaccine without also discussing autism. It is likely that we will be unable to raise vaccination coverage without addressing autism in a way that is satisfactory to Somali mothers. Mothers need to feel that their concerns are taken seriously. Previous studies show inadequate health literacy is also an issue for Somali women [12]. Improving communication and information to parents may in turn change the trend in vaccination. Following this study, we will advise the national and local health authorities to engage with the Somali communities and their trusted figures in Norway to address these issues.

## Conclusion

We have found that children born to Somali parents in Norway have increasingly lower measles vaccination coverage than the national average. The capital and the surrounding county had the lowest coverage and since these cities have large Somali populations, this increases the risk for an outbreak in these locations. In addition, coverage does not seem to improve with the mother’s length of residency, leading us to believe coverage may decrease even more in the future unless appropriate action is taken. Somali boys are particularly at risk.

The Somali diaspora is a world-wide network. Local communities should not be assumed to be independent and isolated from each other. In order to develop new strategies to improve measles vaccine coverage, it is important to study this transnational network. A global conversation is affecting Norway locally, therefore our local strategies need to have a global impact. An aspect of this conversation involves the relationship between MMR and autism, and Somali concerns must be taken seriously. In addition, we need to cooperate with other localities globally for our voice to be heard in this global conversation.

## Data Availability

The datasets generated and/or analysed during the current study are not publicly available due to regulations in the Norwegian Health Research Act and the Norwegian Data Protection Act for use (and storage) of Personal Data related to health, but are available from the corresponding author on reasonable request. To receive access to the data the applicant will need to provide an ethical approval from their IRB or equivalent body, and an exemption from the duty of confidentiality from Health Registry controllers in Norway.

## Abbreviations

MMR vaccine: Measles, mumps and rubella vaccine
SYSVAK: Norwegian Immunisation Registry
UK: United Kingdom
US: United States of America
